# Supplementary material for: The Impact of COPD on Hospitalized Patients with Diabetes: A Propensity Score Matched Analysis on Discharge Records
Source: Healthcare (Basel). 2022 May 11;10(5):885. doi: 10.3390/healthcare10050885 (PMC9140845; doi:10.3390/healthcare10050885)
Supplement: Supplementary file 1 [file healthcare-10-00885-s001.zip › healthcare-1688176-supplementary.pdf]

Supplementary Material

Table S1. Outcomes comparison between diabetic patients with and without COPD by gender

Outcomes	Odds Ratio*	95%CI	p-value
**Males**			
In-hospital mortality	1.10	1.00-1.29	0.047
Lenght of stay over 15 days	1.17	1.11-1.25	<0.001
**Females**			
In-hospital mortality	1.23	1.10-1.43	0.005
Lenght of stay over 15 days	1.10	1.02-1.18	0.020
